# Association of Serum Folate Levels With Cardiovascular Mortality Among Adults With Rheumatoid Arthritis

**DOI:** 10.1001/jamanetworkopen.2020.0100

**Published:** 2020-02-26

**Authors:** Kalyani Sonawane, Yenan Zhu, Wenyaw Chan, David Aguilar, Ashish A. Deshmukh, Maria E. Suarez-Almazor

**Affiliations:** 1 Center for Healthcare Data, Department of Management, Policy, and Community Health, School of Public Health, University of Texas Health Science Center at Houston; 2 Center for Health Services Research, Department of Management, Policy, and Community Health, School of Public Health, University of Texas Health Science Center at Houston; 3 Department of Management, Policy, and Community Health, University of Texas Health Science Center at Houston; 4 Department of Biostatistics and Data Science, University of Texas Health Science Center at Houston; 5 Department of Medicine, Division of Cardiology, University of Texas Health Medical School at Houston; 6 Department of Epidemiology, Human Genetics and Environmental Sciences, University of Texas Health Science Center at Houston; 7 Department of General Internal Medicine, The University of Texas MD Anderson Cancer Center, Houston

## Abstract

**Importance:**

Patients with rheumatoid arthritis (RA) are at high risk for cardiovascular (CV) mortality, attributed to chronic inflammation coupled with elevated circulatory homocysteine levels. Increasing the serum folate level reduces homocysteine, but the association of serum folate concentration with CV mortality in patients with RA has not been previously examined.

**Objective:**

To examine the association of serum folate concentration and CV mortality risk among patients with RA.

**Design, Setting, and Participants:**

A cohort study of the third National Health and Nutrition Examination Survey (1988-1994) and 2011 Linked Mortality File was performed. Adults aged 18 years or older with self-reported physician-diagnosed RA were included. Data analysis was performed between April 2019 and June 2019.

**Exposure:**

Serum folate level.

**Main Outcomes and Measures:**

All-cause and CV mortality risk estimated using Cox proportional hazards models, adjusted for the complex survey design and patient characteristics, including demographic characteristics, body mass index, C-reactive protein level, smoking, RA medication use, and comorbid conditions.

**Results:**

A total of 683 patients with RA (mean [SE] age, 55.9 [1.0] years; 225 [30.2%] men; 478 [87.0%] white) were classified into tertiles based on serum folate levels, as follows: tertile 1, folate levels less than 4.3 ng/mL (n = 239); tertile 2, folate levels 4.3 ng/mL to 8.2 ng/mL (n = 234); and tertile 3, folate levels greater than 8.2 ng/mL (n = 210). During a median (interquartile range) follow-up of 17.4 (10.0-19.4) years, a total of 392 all-cause deaths and 258 CV deaths occurred. Compared with tertile 1, patients in tertile 2 had lower all-cause mortality risk (hazard ratio [HR], 0.63; 95% CI, 0.47-0.85). The risk of CV mortality was lower among patients in tertile 2 (HR, 0.52; 95% CI, 0.30-0.92) and tertile 3 (HR, 0.44; 95% CI, 0.26-0.75) compared with those in tertile 1 (*P* for trend = .01). Findings for CV mortality were consistent in a sensitivity analysis that estimated 10-year risk; patients in tertile 2 (HR, 0.31; 95% CI, 0.17-0.57) and tertile 3 (HR, 0.39; 95% CI, 0.22-0.69) had lower CV mortality risk compared with those in tertile 1 (*P* for trend = .04).

**Conclusions and Relevance:**

Among patients with RA, a serum folate level of at least 4.3 ng/mL was associated with lower CV mortality risk. Further research is needed to examine whether a causal relationship exists between serum folate and CV risk among patients with RA.

## Key Points

**Question:**

Are serum folate levels associated with cardiovascular mortality risk among adults with rheumatoid arthritis (RA)?

**Findings:**

In this cohort study including 683 patients with RA, those with serum folate levels between 4.3 and 8.2 ng/mL and greater than 8.2 ng/mL had 48% and 56% lower cardiovascular mortality risk, respectively, compared with patients who had serum folate levels less than 4.3 ng/mL after adjusting for demographic characteristics, body mass index, C-reactive protein level, smoking, RA medication use, and comorbid conditions.

**Meaning:**

In this study, serum folate levels of at least 4.3 ng/mL were associated with lower cardiovascular mortality risk among patients with RA.

## Teaser

This cohort study examines the association of serum folate concentration with cardiovascular mortality risk among patients with rheumatoid arthritis.

## Introduction

The risk of cardiovascular (CV) mortality among individuals with rheumatoid arthritis (RA) is 60% greater than among the general population.^1^ The etiological reason for increased CV mortality among patients with RA remains unexplained; however, a possible explanation is that increased homocysteine (an established CV risk factor)^2,3,4^ resulting from chronic systemic inflammation may potentially lead to increased CV mortality.^5,6,7,8^ Folic acid, or folate, is an essential nutrient that has a homocysteine-lowering effect. Folate deficiency is common among patients with autoimmune diseases, including RA,^9,10,11^ and it is also a well-documented adverse effect of the disease-modifying antirheumatic drug methotrexate.^10,12^ To counteract reduced folate levels, folate supplements are recommended to patients with RA.^13,14,15,16^ Among patients with RA, an inverse association between serum folate level and homocysteine has been reported.^17^ However, to our knowledge, no published study has examined the association of serum folate levels with long-term CV outcomes. The objective of this study was to determine the association of serum folate levels with CV mortality among patients with RA. Using the third National Health and Nutrition Examination Survey, 1988 to 1994 (NHANES III) and linked mortality data, we examined whether optimal serum folate levels were associated with lower CV mortality risk in adults with RA.

## Methods

### Data Source and Study Design

This is a cohort study of the NHANES III, which contains information from 33 994 US residents aged 2 months or older. The NHANES is a complex, stratified, multistage probability sample of noninstitutionalized US civilians. The survey collects information on demographic characteristics, socioeconomic status, and health conditions and behaviors, which are administered through a personal or telephone interview. Physiologic, dental, and laboratory examinations are included in the survey; these are conducted by trained medical professionals in mobile examination centers (MECs). A detailed description of the NHANES is available elsewhere.^18^ For this study, we merged the NHANES III with the 2011 National Center for Health Statistics Linked Mortality Files. The institutional review board of the University of Texas Health Science Centre at Houston deemed this study exempt from review and informed consent because it uses publicly available deidentified data. This report followed the Strengthening the Reporting of Observational Studies in Epidemiology (STROBE) reporting guideline.

### Cohort Identification

We identified adults aged 18 years or older with a self-reported diagnosis of RA. Self-reported data on medical conditions were collected at the MEC through a standardized questionnaire. Participants were asked, “Has a doctor ever told you that you have arthritis?” Those who responded yes were further asked, “Which type of arthritis was it?” Our cohort was restricted to participants who responded that they were diagnosed with rheumatoid arthritis. We excluded participants who were pregnant during the interview and those with missing data.

### Specimen Collection and Laboratory Methods

Blood specimens of participants were collected in the MEC by trained laboratory technicians, and frozen specimens were shipped overnight per protocol. The NHANES III examined both serum and red blood cell folate values. We used the serum folate value because it is influenced by fewer analytical variables and it shows a higher correlation with homocysteine compared with red blood cell folate.^19,20^ Serum folate levels were measured using the Bio-Rad Laboratories Quantaphase Folate radioassay kit. The assay consists of the preparation of labeled 12 × 75 mm tubes in duplicate for each blank, standard, control, and patient sample. Standard manual procedures, as outlined by the manufacturer for the instrument, were followed. Reportable values for serum folate ranged from 0.4 to 199.0 ng/mL (to convert to nanomoles per liter, multiply by 2.266).

We also evaluated C-reactive protein (CRP) levels as a biomarker for inflammation. The CRP assay was performed on a Behring nephelometer analyzer system. Samples were centrifuged at 3000 rpm for 15 minutes and transferred to a fully automated nephelometer analyzer. Results were automatically printed and calculated by the nephelometer analyzer when the tests were completed. Reportable values for CRP in the NHANES III were between 0.03 mg/L to 2.52 mg/L (to convert to nanomoles per liter, multiply by 9.524). Participants with undetectable values were labeled accordingly. The NHANES III defined normal CRP values as 0 mg/dL to 0.1 mg/L in normal healthy adults based on in-house testing of a sample of 300 patients. For this study, we classified CRP level into the 3 following categories: undetectable (<0.03 mg/L), 0.03 mg/L to 0.1 mg/L, and greater than 0.1 mg/L.

Serum homocysteine (HCYS) levels were analyzed using residual serum samples. The samples were reduced using tributylphosphine to disassociate HCYS from nonrelevant proteins and other disulfides, followed by acid precipitation and the addition of 7-fluorobenzo-2-oxa-1,3-diazole-4-sulfonic acid, a fluorescent reagent. Subsequently, reverse-phase high-performance liquid chromatography and fluorescence detection were used to quantify HCYS. Values for HCYS in the NHANES III ranged from 0.27 mg/L to 17.8 mg/L (to convert to micromoles per liter, multiply by 7.397).

### Demographic and Clinical Characteristics

Demographic data, including age at the time of the interview, sex, and race/ethnicity, were collected through a standard questionnaire administered in-home by trained interviewers using a computer-assisted personal interviewing system. Body mass index (calculated as weight in kilograms divided by height in meters squared) was calculated for each participant based on body measurements collected at the MEC. A detailed questionnaire regarding tobacco use was also administered. We identified participants as current smokers if they responded yes to the question, “Do you smoke cigarettes now?” Hypertension, diabetes, and cardiovascular diseases (CVDs) were also self-reported. For the purpose of this study, we identified participants with CVD as those who responded yes to the questions, “Has a doctor ever told you that you had congestive heart failure?” or “Has a doctor ever told you that you had stroke?” or “Has a doctor ever told you that you had heart attack?” A similar case definition for CVD was used in a previously published report.^21^

### Prescription Drug and Dietary Supplement Use

During the survey, participants were asked if they had taken any prescription drugs and dietary supplements during the past 30 days. Participants who answered yes provided containers of medications and dietary supplements to a trained professional who then matched the product using a prescription drug database (Lexicon Plus; Cerner Multum) and a dietary supplement database (Supliden; National Center for Health Statistics). Information regarding the strength, dosage form, and duration of use was recorded for each medication, provided by the participant. Similarly, information on the dietary blend, strength, and duration of use was recorded for all dietary supplements. We used prescription drug use data to identify patients who reported that they were using disease-modifying antirheumatic drugs, steroids, or nonsteroidal antiinflammatory drugs. Similarly, we used dietary supplement data to identify patients who consumed a dietary supplement containing folic acid.

### Mortality Data

The 2011 National Center for Health Statistics Linked Mortality File consists of mortality information (including mortality status, cause of death, and follow-up time) of NHANES III participants from the date of survey participation until death or December 31, 2011. The Linked Mortality File consists of a leading cause of death variable, which includes 9 cause-specific death categories based on the *International Classification of Diseases, Ninth Revision *(*ICD*-*9*) (through 1999) or *ICD*-*10* (after 1999) code reported on the participant’s death certificate.^22^ We defined all-cause mortality as death because of any reason, while CV mortality included deaths reportedly due to a CVD (ie, leading cause of death code 001 or 005).

### Statistical Analysis

We used descriptive statistics to summarize the characteristics of the entire RA cohort and by tertiles of serum folate level. Frequencies and means were estimated for categorical and continuous variables, respectively. Differences in patient characteristics by serum folate tertiles were tested for statistical significance using an analysis of variance for continuous variables and a χ^2^ test for categorical variables. The distribution of numbers of patients in the 3 CRP categories was determined and compared using a χ^2^ test. Cox proportional hazards models were used to estimate the risk of all-cause and cause-specific (ie, CV) mortality.^23^ The models were adjusted for several potential confounding variables including sex, body mass index, CRP value, and use of steroid or nonsteroid anti-inflammatory drug, based on prior studies.^24,25,26,27,28,29^ We calculated tests of trend with the median folate concentration in each tertile as an ordinal variable. The analyses were repeated for the subgroups of patients without CVD and diabetes. Statistical significance was tested at *P* < .05, and all tests were 2-tailed. All analyses were conducted per the NHANES III analytical guidelines^30^ and were performed with SAS version 9.4 (SAS Institute). We used the survey procedures in SAS, which included weight, cluster, and strata statements, to incorporate sampling weights and to account for the complex survey design. Data analysis was performed between April 2019 and June 2019.

## Results

After applying study criteria, the final cohort included a total of 683 patients with RA (mean [SE] age, 55.9 [1.0] years; 225 [30.2%] men; 478 [87.0%] white); 239 (35.0%) belonged in tertile 1 (ie, folate level <4.3 ng/mL), 234 (34.3%) in tertile 2 (ie, folate level 4.3-8.2 ng/mL), and 210 (30.7%) in tertile 3 (ie, folate level >8.2 ng/mL). The total follow-up duration was 23 years (median [interquartile range], 17.4 [10.0-19.4] years). The study flow is illustrated in eFigure 1 in the Supplement.

### Patient Characteristics

**Figure 1.**
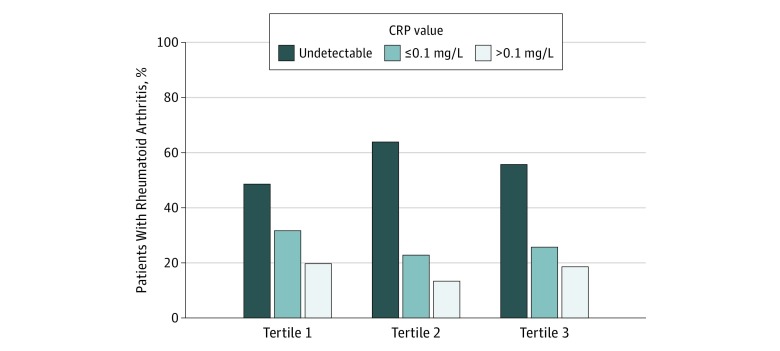
Distribution of C-reactive Protein (CRP) Values in 313 Patients With Rheumatoid Arthritis by Serum Folate Tertile From the Third National Health and Nutrition Survey, 1988-1994 The χ^2^ test value for the difference in the proportion of patients according to CRP was not statistically significant (*P* = .23). The mean CRP values were 1.32 mg/dL among patients in folate tertile 1 (ie, folate levels <4.3 ng/mL), 1.13 mg/dL among patients in folate tertile 2 (ie, folate levels 4.3-8.2 ng/mL), and 1.22 mg/dL among patients in folate tertile 3 (ie, folate levels >8.2 ng/mL). To convert C-reactive protein to nanomoles per liter, multiply by 9.524; to convert folate to nanomoles per liter, multiply by 2.266.

Characteristics of the overall RA cohort and the comparison of patients by folate tertiles are presented in Table 1. Age, race, smoking status, serum HCYS, disease-modifying antirheumatic drug use, and folic acid supplement use were significantly different by serum folate tertiles (eg, mean [SD] age: 52.3 [1.4] years in tertile 1 vs 55.6 [2.0] years in tertile 2 vs 59.4 [1.7] years in tertile 3; *P* = .005; current smokers: 142 [68.0%] in tertile 1 vs 113 [53.2%] in tertile 2 vs 102 [53.4%] in tertile 3; *P* = .04; disease-modifying antirheumatic drug use: 7 [3.5%] in tertile 1 vs 9 [1.9%] in tertile 2 vs 11 [8.0%] in tertile 3; *P* = .03). There was no significant difference in the mean CRP level for 313 patients in detectable range (Table 1), and the distribution of patients in undetectable and detectable ranges of CRP level within each folate tertile was also not significantly different (tertile 1: undetectable CRP levels, 114 [48.6%]; ≤0.1 mg/L, 71 [31.7%]; >0.1 mg/L, 54 [19.7%]; tertile 2: undetectable CRP levels, 141 [63.9%]; ≤0.1 mg/L, 55 [22.8%]; >0.1 mg/L, 38 [13.4%]; tertile 3: undetectable CRP levels, 115 [55.7%]; ≤0.1 mg/L, 62 [25.7%]; >0.1 mg/L, 33 [18.6%]; *P* = .23) (Figure 1).

**Table 1.** Characteristics of Patients With Rheumatoid Arthritis by Serum Folate Tertile From the Third National Health and Nutrition Survey, 1988-1994

Characteristic	No. (%)				*P* Value^b^
	Total (N = 683)	Folate Tertile 1 (n = 239)^a^	Folate Tertile 2 (n = 234)^a^	Folate Tertile 3 (n = 210)^a^	
Age, mean (SE), y	55.86 (1.02)	52.33 (1.36)	55.62 (1.99)	59.42 (1.68)	.005
Men	225 (30.2)	80 (36.8)	82 (31.9)	63 (22.4)	.17
White	478 (87.0)	140 (80.3)	167 (88.0)	171 (92.3)	<.001
Body mass index, mean (SE)^c^	27.88 (0.27)	28.92 (0.53)	27.32 (0.61)	27.45 (0.59)	.14
C-reactive protein, mean (SE), mg/dL^d^	1.23 (0.09)	1.32 (0.17)	1.13 (0.12)	1.21 (0.16)	.62
Serum homocysteine, mean (SE), mg/L^e^	1.34 (0.06)	1.75 (0.09)	1.33 (0.07)	1.08 (0.06)	<.001
Current smoker	357 (58.1)	142 (68.0)	113 (53.2)	102 (53.4)	.04
DMARD use	27 (4.5)	7 (3.5)	9 (1.9)	11 (8.0)	.03
Steroid use	37 (6.0)	15 (7.1)	9 (3.1)	13 (7.9)	.25
NSAID use	171 (23.9)	57 (27.9)	61 (21.0)	53 (23.0)	.25
Folic acid supplement use	179 (32.2)	29 (11.7)	33 (17.8)	117 (65.3)	<.001
Cardiovascular disease	131 (14.8)	43 (14.8)	43 (12.5)	45 (17.0)	.63
Diabetes	107 (12.5)	24 (9.8)	37 (11.2)	46 (15.9)	.23
Hypertension	301 (37.3)	100 (39.1)	101 (38.7)	100 (34.3)	.65

Abbreviations: DMARD, disease-modifying antirheumatic drug; NSAID, nonsteroidal anti-inflammatory drug.

SI conversion factors: To convert C-reactive protein to nanomoles per liter, multiply by 9.524; homocysteine to micromoles per liter, multiply by 7.397; folate to nanomoles per liter, multiply by 2.266.

^a^ Tertile 1 defined as patients with folate levels less than 4.3 ng/mL; tertile 2, 4.3 to 8.2 ng/mL; and tertile 3, greater than 8.2 ng/mL.

^b^ *P* value represents National Health and Nutrition Examination Survey weight-adjusted analysis of variance for continuous variables and χ^2^ test for categorical variables.

^c^ Body mass index was calculated as weight in kilograms divided by height in meters squared.

^d^ Mean values for 313 patients in detectable range for laboratory assay.

^e^ Mean values for 355 patients with nonmissing data for serum homocysteine.

### All-Cause and Cardiovascular Mortality

**Figure 2.**
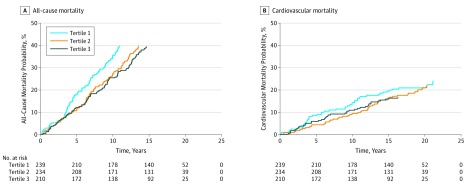
Cumulative Incidence Curves for All-Cause and Cardiovascular Mortality Risk Among Patients With Rheumatoid Arthritis by Serum Folate Tertile From the Third National Health and Nutrition Survey, 1988-1994 Cumulative incidence curves were adjusted for age, sex, race, body mass index, C-reactive protein value, smoking status, disease-modifying antirheumatic drug use, steroid use, nonsteroidal anti-inflammatory drug use, and existing hypertension, diabetes, and cardiovascular disease diagnoses. Tertile 1 includes patients with folate levels less than 4.3 ng/mL; tertile 2, 4.3 to 8.2 ng/mL; and tertile 3, greater than 8.2 ng/mL. To convert folate to nanomoles per liter, multiply by 2.266.

A total of 392 all-cause and 258 CV deaths occurred during the follow-up period. The unadjusted and adjusted results for all-cause and CV mortality risk are presented in Table 2. The risk of all-cause mortality was significantly lower among patients with RA in tertile 2 compared with those in tertile 1 (adjusted hazard ratio [aHR], 0.63; 95% CI, 0.47-0.85). The risk of CV mortality was significantly lower among patients in tertile 2 vs tertile 1 (aHR, 0.52; 95% CI, 0.30-0.92) and among patients in tertile 3 vs tertile 1 (aHR, 0.44; 95% CI, 0.26-0.75) (*P* for trend = .01). The cumulative incidence curves are presented in Figure 2. Factors associated with all-cause and CV mortality in patients with RA are presented in eTable 1 in the Supplement.

**Table 2.** Mortality Risk Among Patients With Rheumatoid Arthritis by Serum Folate Tertile From the Third National Health and Nutrition Examination Survey, 1988-1994

Overall	Folate Tertile 1^a^	Folate Tertile 2^a^	Folate Tertile 3^a^	*P* for Trend
All-cause mortality				
No. with events/No. at risk (%)	121/239 (50.6)	129/234 (55.1)	142/210 (67.6)	NA
Unadjusted HR (95% CI)	1 [Reference]	0.81 (0.56-1.17)	1.13 (0.83-1.54)	.82
Adjusted HR (95% CI)^b^	1 [Reference]	0.63 (0.47-0.85)	0.74 (0.54-1.03)	.99
Cardiovascular mortality				
No. with events/No. at risk (%)	81/239 (33.9)	81/234 (34.6)	96/210 (45.7)	NA
Unadjusted HR (95% CI)	1 [Reference]	0.78 (0.41-1.51)	0.78 (0.44-1.40)	.99
Adjusted HR (95% CI)^b^	1 [Reference]	0.52 (0.30-0.92)	0.44 (0.26-0.75)	.01

Abbreviations: HR, hazard ratio; NA, not applicable.

^a^ Tertile 1 includes patients with folate levels less than 4.3 ng/mL; tertile 2, 4.3 to 8.2 ng/mL; and tertile 3, greater than 8.2 ng/mL (to convert to nmol/L, multiply by 2.266). Models were adjusted for National Health and Nutrition Examination Survey weights.

^b^ Hazard ratio adjusted for age, sex, race, C-reactive protein value, body mass index, smoking, disease-modifying antirheumatic drug use, steroid use, nonsteroidal anti-inflammatory drug use, and history of hypertension, diabetes, and cardiovascular disease.

To examine the robustness of the mortality risk estimates, we conducted a sensitivity analysis by restricting patient follow-up to 10 years. Findings of the sensitivity analysis were consistent with our main analysis. All-cause mortality risk was significantly lower among patients with RA in tertile 2 vs tertile 1 but not among patients in tertile 3 vs tertile 1 (tertile 2: aHR, 0.60; 95% CI, 0.37-0.97; tertile 3: aHR, 0.66; 95% CI, 0.41-1.07; *P* for trend = .20). Patients with RA in tertile 2 (aHR, 0.31; 95% CI, 0.17-0.57) and tertile 3 (aHR, 0.39; 95% CI, 0.22-0.69) had significantly lower CV mortality risk than those in tertile 1 (*P* for trend = .04) (eTable 2 in the Supplement).

### Subgroup Analyses

The main analyses were repeated in the subgroup of patients with RA but no CVD at baseline (Table 3). Compared with patients in tertile 1, all-cause mortality risk was lower for patients with RA in tertile 2 (aHR, 0.48; 95% CI, 0.33-0.71) and tertile 3 (aHR, 0.72; 95% CI, 0.54-0.96; *P* for trend = .42), but the test for trend was not significant. Similarly, the risk of CV mortality was lower for patients with RA in tertile 2 (aHR, 0.42; 95% CI, 0.22-0.78) and tertile 3 (aHR, 0.44; 95% CI, 0.25-0.80) compared with those in tertile 1, but the test for trend was not significant (*P* for trend = .051). Results for the subgroup of RA patients without diabetes at baseline are also presented in Table 3. All-cause mortality risk was lower for patients in tertile 2 vs tertile 1 (aHR, 0.64; 95% CI, 0.45-0.92) than among patients in tertile 3 vs tertile 1 (aHR = 0.72; 95% CI, 0.53-0.99) (*P* for trend = .17). The risk of CV mortality was lower among patients in tertile 3 vs tertile 1 (aHR = 0.48; 95% CI, 0.25-0.91) (*P* for trend = .04). Comparisons of characteristics by serum folate tertile for the RA subgroups without CVD and diabetes are presented in eTable 3 and e Table 4 in the Supplement, respectively. The cumulative incidence curves for all-cause and CV mortality in these RA subgroups are illustrated in eFigure 2 and eFigure 3 in the Supplement.

**Table 3.** Mortality Risk Among Patients With Rheumatoid Arthritis but No CVD or Diabetes From the Third National Health and Nutrition Survey, 1988-1994

Subgroup	HR (95% CI)			*P* for Trend
	Folate Tertile 1^a^	Folate Tertile 2^a^	Folate Tertile 3^a^	
No CVD				
All-cause mortality^b^	1 [Reference]	0.48 (0.33-0.71)	0.72 (0.54-0.96)	.42
CV mortality^b^	1 [Reference]	0.42 (0.22-0.78)	0.44 (0.25-0.80)	.051
No diabetes				
All-cause mortality^c^	1 [Reference]	0.64 (0.45-0.92)	0.72 (0.53-0.99)	.17
CV mortality^c^	1 [Reference]	0.64 (0.34-1.19)	0.48 (0.25-0.91)	.04

Abbreviations: CV, cardiovascular; CVD, cardiovascular disease; HR, hazard ratio.

^a^ Tertile 1 includes patients with folate levels less than 4.3 ng/mL; tertile 2, 4.3 to 8.2 ng/mL; and tertile 3, greater than 8.2 ng/mL (to convert to nmol/L, multiply by 2.266). Folate levels were derived from all eligible participants including those with CVD or diabetes. Models were adjusted for National Health and Nutrition Examination Survey weights.

^b^ Hazard ratio for subgroup of 552 patients without CVD at baseline was adjusted for age, sex, race, C-reactive protein value, body mass index, smoking, disease-modifying antirheumatic drug use, nonsteroidal anti-inflammatory drug use, steroid use (all-cause mortality model only), and history of hypertension and diabetes.

^c^ Hazard ratio for subgroup of 576 patients without diabetes at baseline was adjusted for age, sex, race, C-reactive protein value, body mass index, smoking, disease-modifying antirheumatic drug use, nonsteroidal anti-inflammatory drug use, steroid use, and history of hypertension and CVD.

## Discussion

To our knowledge, our study was the first to examine the association of serum folate level with CV mortality risk among adults with RA. We found that patients with RA and serum folate levels less than 4.3 ng/mL had nearly 50% higher CV mortality risk compared with those with serum folate levels of at least 4.3 ng/mL. Data specific to the RA population are unavailable; however, 2 previous studies of the general, non-RA population have reported increased CV risk with low serum folate levels.^31,32^ In the study by Morrison et al,^31^ participants with serum folate levels less than 3.0 ng/mL had higher risks of coronary heart disease compared with those with serum folate levels greater than 6.0 ng/mL (relative risk, 1.69; 95% CI, 1.10-2.61). Similarly, Loria et al^32^ reported an increased CV mortality risk among participants with serum folate levels less than 4.2 ng/mL compared with those with serum folate levels greater than 7.4 ng/mL (relative risk, 2.64; 95% CI, 1.15-6.09). Furthermore, in a previously published study, patients with RA using supplements containing folate had lower odds of CVD compared with those not receiving folate supplementation (odds ratio, 0.15; 95% CI, 0.06-0.42).^33^ The diagnosis of CVD is an intermediate end point, unlike mortality. Nonetheless, the lower odds of CVD corroborates the findings of the current study, ie, higher serum folate levels were associated with lower CV mortality risk.

Several studies have found an association between folate supplementation, circulating folate concentration, and CVD. The literature on serum folate supplementation provides indirect evidence of the association of serum folate level with CV mortality risk. While a 2006 meta-analysis^34^ reported no CVD or all-cause mortality benefits of folate supplementation in patients with history of vascular disease, a 2016 meta-analysis^35^ reported a 4% lower risk of CVD among patients with preexisting CV or renal diseases. The heterogeneity of cohorts may have led to the discrepancies in CV outcomes observed in these studies. We conducted a subgroup analysis restricted to participants without CVD and diabetes to examine effect modification in our study. Regardless of preexisting CVD or diabetes, patients with RA and serum folate levels less than 4.3 ng/mL in our study had a high risk of CV mortality. Our findings suggest that serum folate levels are associated with CV risk among patients with RA regardless of existing diabetes and CVD.

It is unclear why higher folic acid concentrations would be associated with lower CV mortality risk among patients with RA. A possible explanation is that serum folate lowers CV risk through HCYS reduction. Serum folate levels and plasma homocysteine have an inverse relationship.^36,37^ Empirical studies have reported a successful reduction in HCYS levels among patients with RA supplemented with folic acid. In a multicenter study of patients with RA, plasma HCYS was significantly lower among patients receiving folate supplementation compared with placebo (difference, −0.4 mg/L; *P* < .001).^15^ Two other studies reported an inverse association of serum folate with plasma HCYS in patients with RA.^38,39^ Consistent with these findings, HCYS concentration decreased with increasing folate level in our study. However, it is important to note that we did not observe a dose-response association for serum folate and CV mortality risk. In general, HCYS levels above 1.4 mg/L are considered elevated, and CV risk has been shown to increase in a linear fashion for HCYS levels greater than this threshold.^6,40,41^ Because patients in both tertile 2 (HCYS reduction of 1.3 mg/L) and tertile 3 (HCYS reduction of 1.1 mg/L) had achieved HCYS reductions of less than 1.4 mg/L, CV risk estimates for these serum tertiles were not statistically significantly different. The lack of dose-response association for serum folate and CV mortality is consistent with findings in the general population.^32^

### Limitations

This study has limitations. The diagnosis of RA was self-reported in our study. The case definition for arthritis (ie, self-reported physician-diagnosed arthritis) used in our study is recommended by the US Centers for Disease Control and Prevention and has been validated.^42,43^ It is possible that limitations of survey methodology (ie, information was collected only on prescription drugs that were used by survey participants within 30 days before the interview) may have led to lower number of treated patients with RA in NHANES III; however, incorporation of drug information is recommended, and previous studies also have reported poor treatment rates in RA.^44,45^ The folate status of patients was based on a single serum folate concentration measurement that may not accurately represent long-term folate levels; however, pathological studies have shown positive correlation (*R* = 0.53) between serum folate and liver folate (a laboratory biomarker for tissue stores of folate).^46^ Next, consistent with findings in the general population,^32^ estimates for all-cause mortality were not significant. All-cause mortality, by definition, includes deaths due to any non-CV cause (ie, accidental or injury-related death or death due to other health conditions) and presumably has no association with serum folate. Although most confounders were taken into consideration, we did not adjust our analyses for factors that might affect serum folate levels, including health conditions in which folate absorption lowers (eg, celiac diseases or Crohn disease) or use of drugs such as phenytoin. Furthermore, the observational nature of the data precludes causal inference, and the timeframe of NHANES III data collection limits the generalizability of our findings to current clinical practices.

## Conclusions

Our findings suggest that serum folate level is associated with CV mortality risk among patients with RA and might be a useful indicator for assessing patient risk in clinical practice. Additionally, if a causal link is validated in future clinical studies, folate supplementation can be an inexpensive strategy for reducing CV mortality risk in patients with RA.

## Notes

Supplement. **eTable 1. **Factors Associated With All-Cause and Cardiovascular Mortality in Rheumatoid Arthritis, NHANES III **eTable 2.** Ten-Year Mortality Risk in Patients with Rheumatoid Arthritis by Serum Folate Tertile, NHANES III **eTable 3.** Characteristics of Patients With Rheumatoid Arthritis but Without Cardiovascular Disease by Folate Tertile, NHANES III **eTable 4.** Characteristics of Patients With Rheumatoid Arthritis but Without Diabetes by Folate Tertile, NHANES III **eFigure 1. **Sample Flow Diagram **eFigure 2. **Cumulative Incidence Curves for All-Cause and Cardiovascular Mortality Risk in Patients With Rheumatoid Arthritis but Without Cardiovascular Disease by Serum Folate Tertile, NHANES III **eFigure 3. **Cumulative Incidence Curves for All-Cause and Cardiovascular Mortality Risk in Patients With Rheumatoid Arthritis but Without Diabetes by Serum Folate Tertile, NHANES III
