# Supplementary material for: Association of Serum Folate Levels With Cardiovascular Mortality Among Adults With Rheumatoid Arthritis
Source: JAMA Netw Open. 2020 Feb 26;3(2):e200100. doi: 10.1001/jamanetworkopen.2020.0100 (PMC7137687; doi:10.1001/jamanetworkopen.2020.0100)
Supplement: Supplement. — eTable 1. Factors Associated With All-Cause and Cardiovascular Mortality in Rheumatoid Arthritis, NHANES III eTable 2. Ten-Year Mortality Risk in Patients with Rheumatoid Arthritis by Serum Folate Tertile, NHANES III eTable 3. Characteristics of Patients With Rheumatoid Arthritis but Without Cardiovascular Disease by Folate Tertile, NHANES III eTable 4. Characteristics of Patients With Rheumatoid Arthritis but Without Diabetes by Folate Tertile, NHANES III eFigure 1. Sample Flow Diagram eFigure 2. Cumulative Incidence Curves for All-Cause and Cardiovascular Mortality Risk in Patients With Rheumatoid Arthritis but Without Cardiovascular Disease by Serum Folate Tertile, NHANES III eFigure 3. Cumulative Incidence Curves for All-Cause and Cardiovascular Mortality Risk in Patients With Rheumatoid Arthritis but Without Diabetes by Serum Folate Tertile, NHANES III [file jamanetwopen-3-e200100-s001.pdf]

# Supplementary Online Content

Sonawane K, Zhu Y, Chan W, Aguilar D, Deshmukh AA, Suarez-Almazor ME. Association of serum folate levels with cardiovascular mortality among adults with rheumatoid arthritis. *JAMA Netw Open*. 2020;3(2):e200100. doi:10.1001/jamanetworkopen.2020.0100

**eTable 1.** Factors Associated With All-Cause and Cardiovascular Mortality in Rheumatoid Arthritis, NHANES III
**eTable 2.** Ten-Year Mortality Risk in Patients with Rheumatoid Arthritis by Serum Folate Tertile, NHANES III
**eTable 3.** Characteristics of Patients With Rheumatoid Arthritis but Without Cardiovascular Disease by Folate Tertile, NHANES III
**eTable 4.** Characteristics of Patients With Rheumatoid Arthritis but Without Diabetes by Folate Tertile, NHANES III
**eFigure 1.** Sample Flow Diagram
**eFigure 2.** Cumulative Incidence Curves for All-Cause and Cardiovascular Mortality Risk in Patients With Rheumatoid Arthritis but Without Cardiovascular Disease by Serum Folate Tertile, NHANES III
**eFigure 3.** Cumulative Incidence Curves for All-Cause and Cardiovascular Mortality Risk in Patients With Rheumatoid Arthritis but Without Diabetes by Serum Folate Tertile, NHANES III

This supplementary material has been provided by the authors to give readers additional information about their work.

© 2020 Sonawane K et al. *JAMA Network Open.*

**eTable 1.** Factors Associated With All-Cause and Cardiovascular Mortality in Rheumatoid Arthritis, NHANES III

| CHARACTERISTIC | HAZARD RATIO (95% CI)* | |
|---|---|---|
| | ALL-CAUSE | CARDIOVASCULAR |
| Folate Tertile† | | |
| *III vs. I* | 0.74 (0.54-1.03) | 0.44 (0.26-0.75) |
| *II vs. I* | 0.63 (0.47-0.85) | 0.52 (0.30-0.92) |
| C-reactive Protein | | |
| *>1 mg/dL vs. Undetected* | 1.98 (1.46-2.69) | 1.23 (0.75-2.03) |
| *0.3-1.0 vs. Undetected* | 1.49 (0.97-2.29) | 1.36 (0.61-3.03) |
| Age | 1.09 (1.07-1.11) | 1.09 (1.06-1.13) |
| Female vs. Male | 0.79 (0.54-1.15) | 1.20 (0.55-2.66) |
| White vs. Other | 1.11 (0.85-1.47) | 1.31 (0.84-2.04) |
| Body Mass Index | 1.00 (0.98-1.02) | 0.99 (0.96-1.03) |
| Current smoker (Yes vs. No) | 1.48 (1.18-1.86) | 1.22 (0.77-1.95) |
| Using DMARD (Yes vs. No) | 0.94 (0.51-1.73) | 0.48 (0.06-3.75) |
| Using NSAID (Yes vs. No) | 1.21 (0.71-2.06) | 0.68 (0.38-1.20) |
| Using Steroid (Yes vs. No) | 0.90 (0.69-1.18) | 1.50 (0.51-4.41) |
| Hypertension (Yes vs. No) | 1.08 (0.84-1.39) | 1.23 (0.73-2.04) |
| Diabetes (Yes vs. No) | 2.68 (1.96-3.66) | 2.68 (1.38-5.19) |
| Existing CVD (Yes vs. No) | 1.09 (0.82-1.46) | 1.20 (0.74-1.93) |

NHANES, National Health and Nutrition Examination Survey; CI, Confidence Interval; DMARD, disease-modifying anti-rheumatic drug; NSAID, non-steroidal anti-inflammatory drug, CVD, cardiovascular disease.
*Models were adjusted for NHANES survey weights.
†Folate Tertile cut-off values: 4.3 and 8.2 ng/mL.

© 2020 Sonawane K et al. *JAMA Network Open.*

**eTable 2.** Ten-Year Mortality Risk in Patients with Rheumatoid Arthritis by Serum Folate Tertile, NHANES III

| OVERALL | FOLATE TERTILE* | | | *P* for trend |
|---|---|---|---|---|
| | I | II | III | |
| **ALL CAUSE MORTALITY** | | | | |
| No. with events / No. at risk | 61/239 | 65/234 | 75/210 | |
| Unadjusted HR (95% CI ) | Ref | 0.80 (0.48-1.33 | 1.14 (0.73-1.77) | 0.34 |
| Adjusted HR (95% CI )† | Ref | 0.60 (0.37-0.97) | 0.66 (0.41-1.07) | 0.20 |
| **CARDIOVASCULAR MORTALITY** | | | | |
| No. with events / No. at risk | 26/239 | 23/234 | 30/210 | |
| HR (unadjusted) | Ref | 0.45 (0.22-0.94) | 0.74 (0.42-1.33) | 0.59 |
| 95% CI | Ref | | | |
| HR (adjusted)† | Ref | 0.31 (0.17-0.59) | 0.39 (0.22-0.69) | 0.04 |
| 95% CI | Ref | | | |

NHANES, National Health and Nutrition Examination Survey; HR, Hazard ratio; CI, Confidence Interval.
*Folate Tertile cut-off values: 9.7 and 18.6 nmol/L. Models were adjusted for NHANES survey weights.
†Hazard ratio adjusted for age, sex, race, C-reactive protein value, body mass index, smoking, disease-modifying anti-rheumatic drug use, steroid use, non-steroidal anti-inflammatory drug use, and history of hypertension, diabetes, and cardiovascular disease.

© 2020 Sonawane K et al. *JAMA Network Open.*

**eTable 3.** Characteristics of Patients With Rheumatoid Arthritis but Without Cardiovascular Disease by Folate Tertile, NHANES III

| CHARACTERISTIC | TOTAL | FOLATE TERTILE* | | |
|---|---|---|---|---|
| | | I | II | III |
| Mean Age (SE), in years | 53.72 (1.08) | 50.38 (1.41) | 53.46 (2.07) | 57.22 (1.85) |
| Male, n (%) | 171 (29.59) | 63 (35.78) | 63 (31.09) | 45 (22.08) |
| White, n (%) | 383 (87.25) | 119 (81.42) | 133 (88.21) | 131 (91.93) |
| Mean body mass index (SE) | 27.60 (0.35) | 28.27 (0.53) | 27.29 (0.64) | 27.29 (0.72) |
| Mean C-reactive protein (SE), mg/L | 0.118 (0.010) | 0.128 (0.019) | 0.099 (0.012) | 0.121 (0.018) |
| Current smoker, n (%) | 282 (57.60) | 114 (67.92) | 85 (51.31) | 83 (53.94) |
| DMARD Use, n (%) | 25 (5.2) | 7 (4.1) | 8 (2.1) | 10 (9.4) |
| Steroid Use, n (%) | 28 (6.2) | 11 (6.1) | 5 (3.0) | 12 (9.4) |
| NSAID Use, n (%) | 129 (21.8) | 41 (24.6) | 45 (17.4) | 43 (23.5) |
| Folic Acid Supplement Use, n (%) | 146 (32.1) | 24 (10.9) | 28 (18.8) | 94 (66.1) |
| Diabetes, n (%) | 68 (8.01) | 16 (1.92) | 25 (8.20) | 27 (9.88) |
| Hypertension, n (%) | 217 (32.81) | 71 (35.88) | 78 (35.60) | 68 (27.02) |

NHANES, National Health and Nutrition Examination Survey; SE, Standard error; DMARD, Disease-modifying anti-rheumatic drug; NSAID, Non-steroidal anti-inflammatory drug.
*Folate Tertile cut-off values: 4.3 and 8.2 ng/mL.

© 2020 Sonawane K et al. *JAMA Network Open.*

**eTable 4.** Characteristics of Patients With Rheumatoid Arthritis but Without Diabetes by Folate Tertile, NHANES III

| CHARACTERISTIC | TOTAL | FOLATE TERTILE* | | |
|---|---|---|---|---|
| | | I | II | III |
| Mean Age (SE), in years | 54.46 (1.06) | 51.19 (1.30) | 54.46 (2.05) | 57.76 (1.92) |
| Male, n (%) | 194 (30.81) | 77 (38.77) | 69 (31.06) | 48 (22.49) |
| White, n (%) | 401 (87.37) | 122 (79.45) | 140 (88.72) | 139 (94.03) |
| Mean body mass index (SE) | 27.35 (0.33) | 28.19 (0.49) | 27.05 (0.66) | 26.81 (0.68) |
| Mean C-reactive protein (SE), mg/dL | 0.121 (0.011) | 0.134 (0.019) | 0.111 (0.014) | 0.112 (0.019) |
| Current smoker, n (%) | 301 (57.96) | 131 (69.77) | 94 (52.26) | 76 (51.72) |
| DMARD Use, n (%) | 26 (5.0) | 7 (3.8) | 9 (2.1) | 10 (9.0) |
| Steroid Use, n (%) | 30 (5.9) | 9 (5.0) | 8 (3.3) | 13 (9.4) |
| NSAID Use, n (%) | 128 (19.8) | 47 (26.0) | 46 (15.9) | 35 (17.6) |
| Folic Acid Supplement Use, n (%) | 148 (32.5) | 28 (12.0) | 31 (19.6) | 89 (66.3) |
| Cardiovascular Disease, n (%) | 92 (10.42) | 35 (11.12) | 31 (9.06) | 26 (11.07) |
| Hypertension, n (%) | 234 (34.28) | 81 (34.83) | 79 (36.11) | 74 (31.88) |

NHANES, National Health and Nutrition Examination Survey; SE, Standard error; DMARD, Disease-modifying anti-rheumatic drug; NSAID, Non-steroidal anti-inflammatory drug.
*Folate Tertile cut-off values: 4.3 and 8.3 ng/mL.

© 2020 Sonawane K et al. *JAMA Network Open.*

**eFigure 1.** Sample Flow Diagram

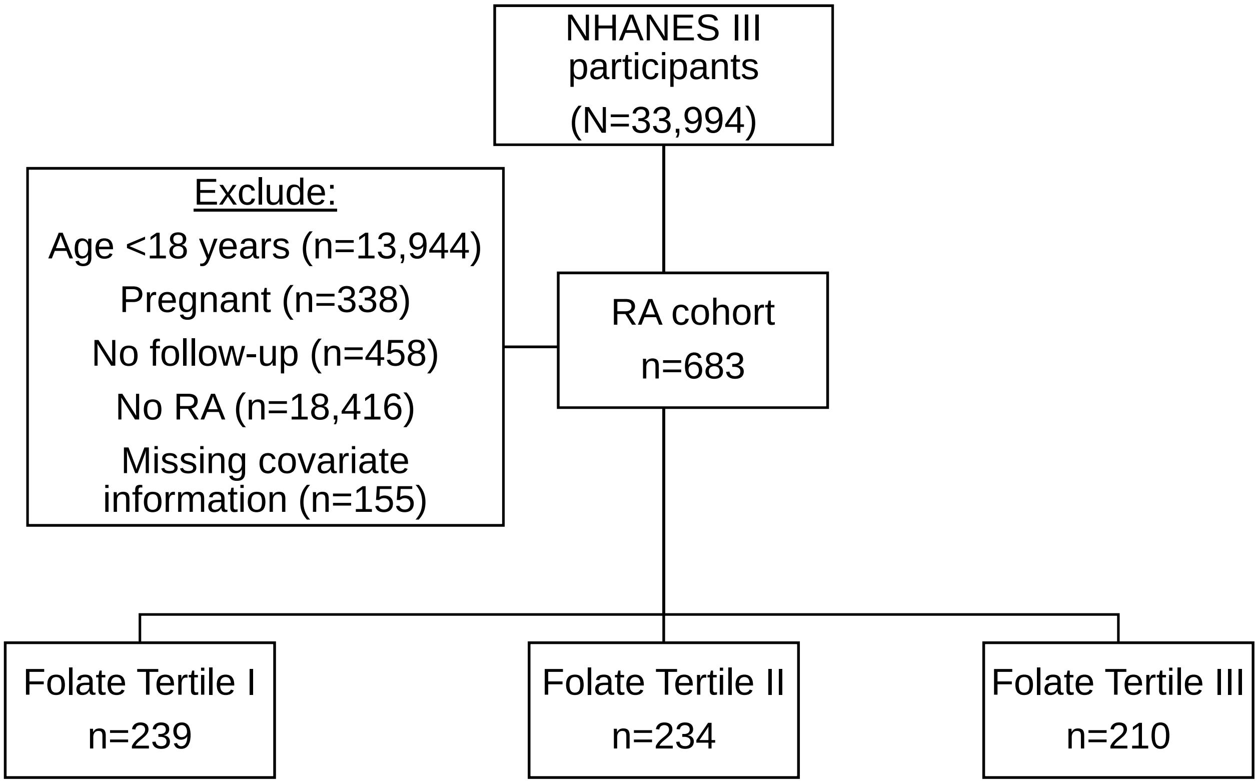


© 2020 Sonawane K et al. *JAMA Network Open.*

**eFigure 2.** Cumulative Incidence Curves for All-Cause and Cardiovascular Mortality Risk in Patients With Rheumatoid Arthritis but Without Cardiovascular Disease by Serum Folate Tertile, NHANES III

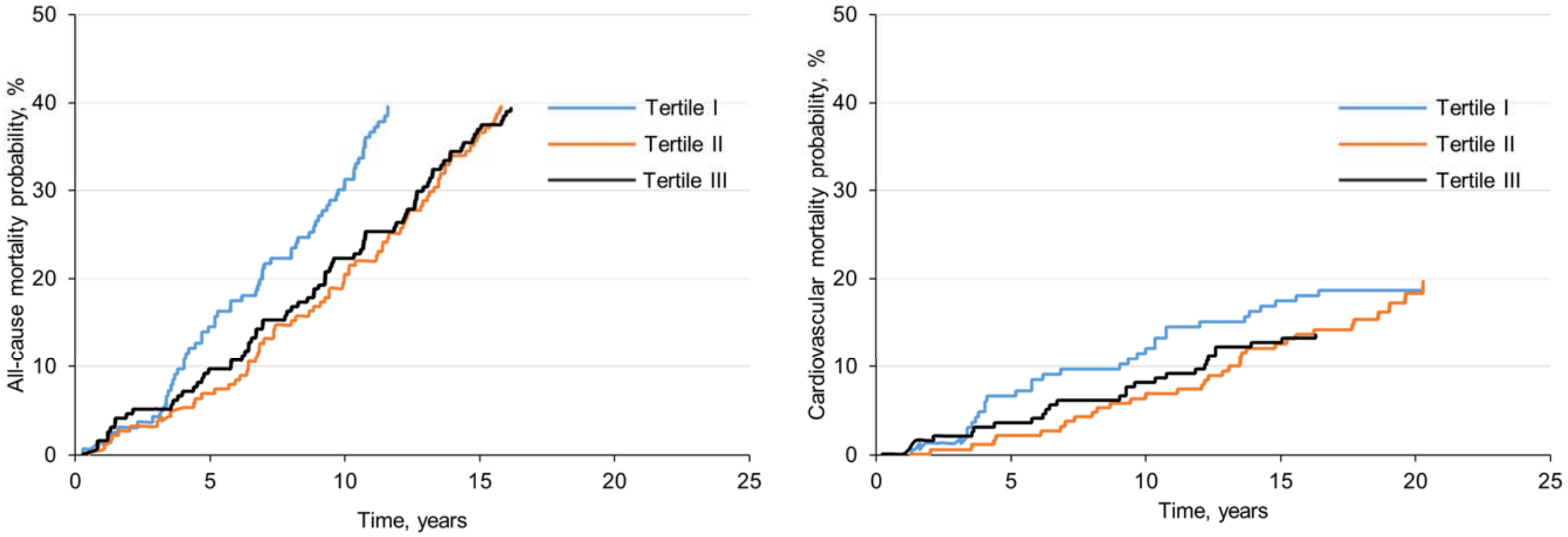


© 2020 Sonawane K et al. *JAMA Network Open.*

**eFigure 3.** Cumulative Incidence Curves for All-Cause and Cardiovascular Mortality Risk in Patients With Rheumatoid Arthritis but Without Diabetes by Serum Folate Tertile, NHANES III

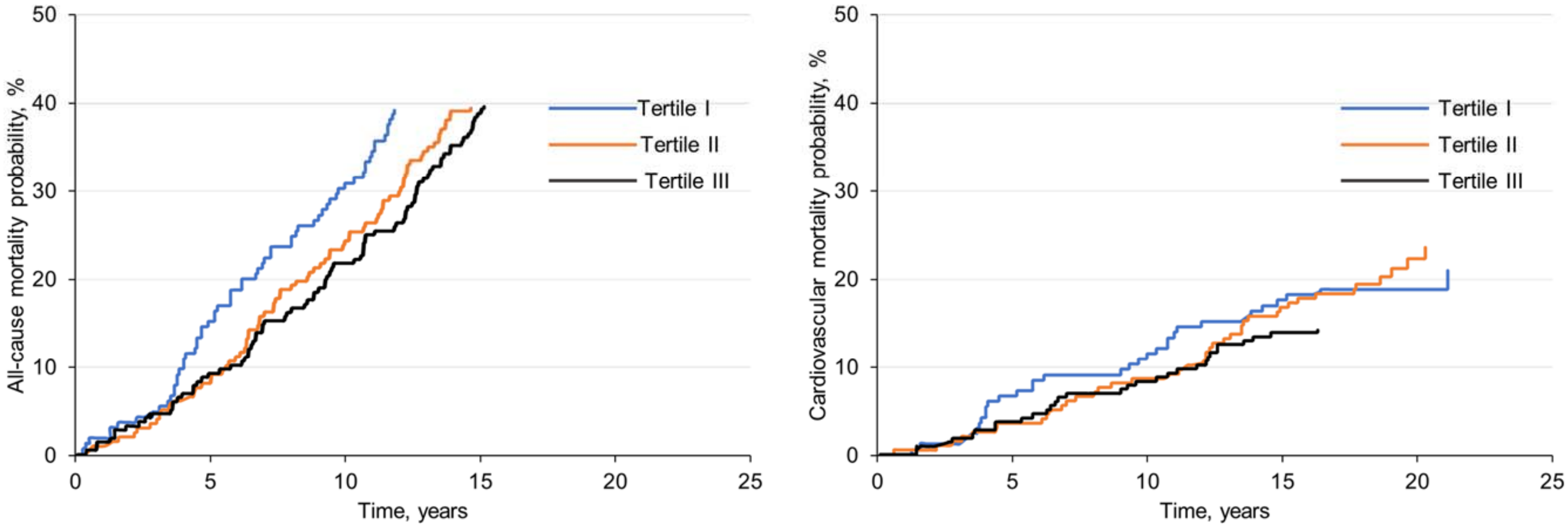


© 2020 Sonawane K et al. *JAMA Network Open.*
